# Synthetic lethality in large-scale integrated metabolic and regulatory network models of human cells

**DOI:** 10.1038/s41540-023-00296-3

**Published:** 2023-07-15

**Authors:** Naroa Barrena, Luis V. Valcárcel, Danel Olaverri-Mendizabal, Iñigo Apaolaza, Francisco J. Planes

**Affiliations:** 1 University of Navarra, Tecnun School of Engineering, Manuel de Lardizábal 13, 20018 San Sebastián, Spain; 2 University of Navarra, Biomedical Engineering Center, Campus Universitario, 31009 Pamplona, Navarra Spain; 3 University of Navarra, Instituto de Ciencia de los Datos e Inteligencia Artificial (DATAI), Campus Universitario, 31080 Pamplona, Spain

**Keywords:** Biochemical networks, Cancer, Software

## Abstract

Synthetic lethality (SL) is a promising concept in cancer research. A wide array of computational tools has been developed to predict and exploit synthetic lethality for the identification of tumour-specific vulnerabilities. Previously, we introduced the concept of genetic Minimal Cut Sets (gMCSs), a theoretical approach to SL developed for genome-scale metabolic networks. The major challenge in our gMCS framework is to go beyond metabolic networks and extend existing algorithms to more complex protein-protein interactions. In this article, we take a step further and incorporate linear regulatory pathways into our gMCS approach. Extensive algorithmic modifications to compute gMCSs in integrated metabolic and regulatory models are presented in detail. Our extended approach is applied to calculate gMCSs in integrated models of human cells. In particular, we integrate the most recent genome-scale metabolic network, Human1, with 3 different regulatory network databases: Omnipath, Dorothea and TRRUST. Based on the computed gMCSs and transcriptomic data, we discovered new essential genes and their associated synthetic lethal for different cancer cell lines. The performance of the different integrated models is assessed with available large-scale in-vitro gene silencing data. Finally, we discuss the most relevant gene essentiality predictions based on published literature in cancer research.

## Introduction

Two (or more) genes are synthetic lethal when the loss of function of either gene on its own is compatible with cell viability, while the co-occurrence of them leads to cellular death^1^. Given the plethora of tumour-specific genetic alterations, synthetic lethality (SL) is an attractive approach to identify selective drug targets in cancer cells. This has propelled the development of robust methods to identify synthetic lethals from very different perspectives^2–6^.

In previous works^7,8^, we introduced the concept of genetic Minimal Cut Sets (gMCSs), a theoretical approach to SL based on genome-scale metabolic networks. gMCSs define minimal set of gene knockouts that blocks a particular metabolic task, typically the biomass reaction in cancer studies. They can be easily integrated with -omics data and used to elucidate metabolic vulnerabilities in cancer cells. Recently, based on data from the Cancer Dependency Map (DepMap)^9,10^, we assessed the capacity of our gMCS approach to predict gene essentiality in cancer cell lines and reported a superior performance than other network-based algorithms^11^. In a different work^12^, we also integrated nutritional perturbations into our gMCS framework, leading to nutrient dependencies in cancer cell lines.

Unfortunately, our current gMCS framework is constrained to the metabolic space, which represents only a fraction of all the interactions that occur within a cell. For instance, the latest reconstruction of human metabolism, Human1^13^, only represents 22% of the genes available in Omnipath^14^, one of the biggest protein-protein interactions database. For this reason, the main challenge of our gMCS approach is to go beyond metabolic networks and extend existing algorithms to more complex protein-protein interactions, such as signalling or regulatory networks.

Our gMCS approach is built on gene-protein-reaction (GPR) rules available in genome-scale metabolic models^15^. A natural way to extend our gMCS formulation is to incorporate regulatory information into these GPR rules, as done in other constraint-based modelling tools^16–18^. However, GPR rules in metabolic models are simple Boolean networks without negation terms and cycles, which are typically present in regulatory networks. This fundamental difference makes particularly challenging the integration of regulatory networks with our gMCS approach, which currently cannot deal with Boolean equations involving negation terms and cycles^8^.

Here, we present the required algorithmic modifications of our previous gMCS formulation to incorporate linear regulatory pathways. Our extended approach is applied to calculate gMCSs in integrated metabolic and regulatory models of human cells. In particular, we consider Human1^13^ with 3 different regulatory network databases: Omnipath^14^, Dorothea^19^ and TRRUST^20^. Based on the computed gMCSs and transcriptomic data, we detail new essential genes and their associated synthetic lethals for different cancer cell lines. The performance of the different integrated models is assessed with available large-scale in vitro gene silencing data^9,10,21^. Finally, we discuss the most relevant gene essentiality predictions based on published literature in cancer research.

## Results

In previous works, we presented different optimisation algorithms to calculate gMCSs in metabolic networks and identify cancer-specific essential genes based on transcriptomic data^7,11^. Here, we extend our previous gMCS formulation to consider integrated networks involving metabolic and linear (acyclic) regulatory pathways. As detailed in the Methods section, we describe how to: (i) build extended GPR (eGPR) rules for different regulatory layers avoiding the presence of cycles; (ii) amend the computation of *G* matrix, a critical component in our gMCS formulation, which defines for each row a subset of reactions deleted by an irreducible subset of gene knockouts; (iii) calculate gMCSs in these integrated metabolic and regulatory models. Moreover, gene essentiality analysis in cancer was modified to consider possible adaptation mechanisms that can be driven by regulatory pathways (see Methods section).

In order to assess the performance of our extended approach, we integrated the large-scale curated and most recently published metabolic network of human cells, Human1^13,22^ (v1.14.0), with the protein-protein interaction network of Omnipath^14^ (v.3.4.7)^23^, the gene regulatory network of signed transcription factors Dorothea^19^ (v.1.7.2) and the manually curated database of human transcriptional regulatory networks TRRUST^20^. We present below the analysis of identified gMCSs for different integrated models with single and multiple-regulatory layers and resulting gene essentiality analysis in cancer cell lines.

### Analysis of gMCSs in single-layer integrated metabolic and regulatory models

First, we built 3 integrated models with a unique layer of regulatory interactions for each metabolic gene: Human1 + Omnipath (Human1-O1); Human1 + Dorothea (Human1-D1), Human1 + TRRUST (Human1-T1). The addition of the regulatory layer increased the number of genes in the 3 cases, being Human1-O1 the one with the highest increase (Table 1). However, we obtained the largest *G* matrix and highest computation time with Human1-D1 (Table 1), which involves more complex Boolean regulatory rules than Human1-O1 and Human1-T1. As partially expected, the computation time scales linearly with the number of rows of *G* matrix (Pearson’s correlation = 0.95, *p* value = 0.02536). Note here that no genetic interactions were lost in these models due to the presence of cycles in eGPR rules (see Methods section).

**Table 1:** Summary of single-layer integrated metabolic and regulatory models and computed gMCSs.

Model	Number of genes	*G* matrix dimension	*G* matrix computation time (s)	*Simplified G matrix*	Number of gMCSs (length ≤ 5)
Human1	2419	1736 × 11,573	563	1577 × 11,573	10,091
Human1-O1	3046	2533 × 11,573	2629	1800 × 11,573	14,060 (3969)
Human1-D1	2517	4173 × 11,573	4061	2024 × 11,573	11,435 (1344)
Human1-T1	2654	2679 × 11,573	1894	1801 × 11,573	10,779 (688)

Computation time is given in seconds (s).

*Human1-O1* integrated model with Human1 and Omnipath with one regulatory layer, *Human1-D1* integrated model with Human1 and Dorothea with one regulatory layer, *Human1-T1* integrated model with Human1 and TRRUST with one regulatory layer. In the column ‘Number of gMCSs (length ≤ 5)’, the number in parenthesis is the number of gMCSs arising from the addition of the regulatory layer.

For each model, we calculated gMCSs until length 5 that block biomass production. To reduce the computation cost, we deleted rows in *G* involving more than 5 genes, leading to the simplified *G* matrix (Table 1), which substantially reduces memory requirements. 10091 gMCSs were identified for Human1 (Table 1). All of them were included in our 3 integrated models (Supplementary Fig. 1); however, we found 5999 new gMCSs: 3969 in Human1-O1, 1344 in Human1-D1 and 688 in Human1-T1 (Table 1). We observed that the new subset of gMCSs identified strongly depends on the regulatory database employed and shows limited overlap (Supplementary Fig. 1).

**Fig. 1:**
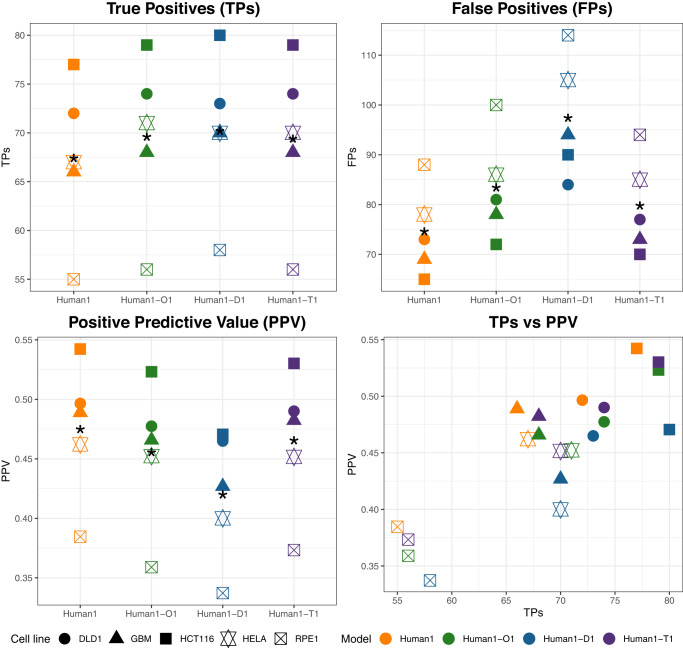
Gene essentiality comparison between Human1 and single-layer integrated metabolic and regulatory models. True Positives (TPs), False Positives (FPs), Positive Predictive Value (PPV) and TPs vs PPV arising from our different models (Human1, Human1-O1, Human-D1, Human1-T1) using the essentiality data presented in Hart2015 as a gold-standard. An asterisk * represents the mean value for the 5 cell lines considered.

Given the differences found in the different integrated models, we compared their capacity to predict essential genes in cancer, following the computational approach described in the Methods section. We used as a gold standard the genome-wide CRISPRi experiments from 5 cancer cell lines published by Hart and colleagues^21^, referred to as Hart2015, and gene expression data from CCLE^9^. Once the list of essential genes per cell line and per integrated model was computed, we compared them with the essentiality scores of Hart2015. We determined the number of true positives (TPs) and false positives (FPs), as well as the positive predictive value (PPV), which is the ratio TPs to all of the genes that were defined as positive (TP + FP) (Fig. 1).

As shown in Fig. 1, the addition of a regulatory layer involves a significant increase in the number of TPs in our three integrated models (paired *t* test *p* value ≤ 0.05). However, FPs also significantly rise (paired *t* test *p* value ≤ 0.05), and, thus, the PPV of the integrated models is slightly lower than in Human1. In particular, Dorothea leads to the detection of more TPs, but it is also the one with the highest value of FPs and, so, the lowest PPV of all the models (average PPV in Human1-D1 = 0.42). TRRUST and Omnipath present a better proportion of TPs and FPs than Dorothea, obtaining a higher average PPV value (Human1-O1 = 0.456, Human1-T1 = 0.465), which is very close to Human1 (Human1 = 0.475). Interestingly, as it is shown in the plot TPs vs PPV, although the PPV value decreases with the addition of the regulatory layer, the integrated models always dominate Human1 in terms of TPs. A similar conclusion was obtained for DepMap data^9,10^ (Supplementary Fig. 2).

Each regulatory database led to the detection of specific subsets of essential genes. For example, in the cell line HELA, we found the same 145 metabolic essential genes in all the models; 30 new essential genes with Human1-D1, among which 4 are shared with Human1-T1 and 1 with Human1-O1; 12 new essential genes with Human1-O1, among which 1 is shared with Human1-D1; and 10 new essential genes in Human1-T1, among which 4 are shared with Human1-D1 (Supplementary Fig. 3). In addition, new essential genes are transcription factors but also metabolic enzymes. An example of this cell line is TXN2. In Human1, it appears in unique gMCSs of length 2: TXN & TXN2. TXN is expressed in HELA, and, for that reason, TXN2 is not predicted as essential in Human1. However, in Human1-T1, TXN2 appears in 2 gMCSs: {TXN2 & TXN}, {TXN2 & PPARD}. In HELA, the gene PPARD is not expressed, and, therefore, TXN2 is predicted as an essential gene.

For completeness, we analysed the impact of the combination of regulatory databases on the accuracy of gene essentiality analysis. First, we assessed the union of different databases (deleting contradictory interactions) and built 4 integrated models: Human1 + the union of Omnipath and Dorothea (Human1-O∪D1), Human1 + the union of Omnipath and TRRUST (Human1-O∪T1), Human1 + the union of Dorothea and TRRUST (Human1-D∪T1), Human1 + the union of Omnipath, Dorothea and TRRUST (Human1-O∪D∪T1). However, none of these integrated models did better than Human1-T1 (average PPV ≤ 0.452, Supplementary Fig. 4). Moreover, we considered the intersection of databases and built 4 additional models: Human1-O ∩ D1, Human1-O ∩ T1, Human1-D ∩ T1, and Human1-O ∩ D ∩ T1. We found a slightly better accuracy than Human1-T1 in all the cases. However, this is due to the limited overlap among databases, which makes the relevance of the regulatory layer very low and integrated models very similar to Human1 (Supplementary Table 1).

### Analysis of gMCSs in multiple-layer integrated metabolic and regulatory models

Table 2 shows the details for the different integrated models including 1, 2 and 3 layers of regulatory interactions for each metabolic gene (see Methods section). The addition of multiple layers has particularly an impact on Human1-O, which involves 4591 genes in the third layer (Human1-O3). In Human1-D and Human1-T, the impact of multiple layers is moderate and the third layer seems irrelevant. Although Dorothea and TRRUST are smaller databases than Omnipath, the presence of cycles in eGPR rules limits the inclusion of a higher number of genes in the third layer. In particular, cycles affect 743 reactions in Human1-T3 and 2644 reactions in Human1-D3. Therefore, the cycle limitation restricts the application of our approach to more than 2 layers in TRRUST and Dorothea. The effect of cycles in Omnipath (1301 reactions affected in Human1-O3) is counteracted by the size of the database (see Table 3 in the Methods section).

**Table 2:** Summary of multiple-layer integrated metabolic and regulatory models and computed gMCSs.

Model	Number of genes	*G* matrix dimension	*G* matrix computation time (s)	Simplified G matrix	Number of gMCSs (length ≤ 5)
Human1	2419	1736 × 11,573	562	1577 × 11,573	10,091
Human1-O1	3046	2533 × 11,573	2629	1800 × 11,573	14,060 (3969)
Human1-O2	4334	42,936 × 11,573	85,155	3118 × 11,573	15,104 (5013)
Human1-O3	4591	45,357 × 11,573	102,506	3630 × 11,573	18,624 (8533)
Human1-D1	2517	4173 × 11,573	4061	2024 × 11,573	11,435 (1344)
Human1-D2	2527	4973 × 11,573	7140	2194 × 11,573	11,248 (1157)
Human1-D3	2527	5068 × 11,573	7464	2242 × 11,573	11,249 (1158)
Human1-T1	2654	2679 × 11,573	1894	1801 × 11,573	10,779 (688)
Human1-T2	2800	6988 × 11,573	4850	2349 × 11,573	10,944 (853)
Human1-T3	2828	17,590 × 11,573	16,021	2708 × 11,573	10,908 (817)

Results correspond to models including 1, 2 and 3 regulatory layers. Computation time is given in seconds (s). In the column ‘Number of gMCSs (length≤5)’, the number in parenthesis is the number of gMCSs arising from the addition of the regulatory layer.

*Human1-O1* integrated model with Human1 and Omnipath with one regulatory layer, *Human1-O2* integrated model with Human1 and Omnipath with two regulatory layers, *Human1-O3* integrated model with Human1 and Omnipath with three regulatory layers, *Human1-D1* integrated model with Human1 and Dorothea with one regulatory layer, *Human1-D2* integrated model with Human1 and Dorothea with two regulatory layers, *Human1-D3* integrated model with Human1 and Dorothea with three regulatory layers, *Human1-T1* integrated model with Human1 plus TRRUST and one regulatory layer, *Human1-T2* integrated model with Human1 plus TRRUST and two regulatory layers, *Human1-T3* integrated model with Human1 and TRRUST with three regulatory layers.

**Table 3:** Description of the main features of the regulatory networks employed in the analysis.

Regulatory network	Number of Interactions	Number of genes	Number of metabolic genes	Number of target metabolic genes
Omnipath	19,964	6044	720	544
Dorothea	6500	3313	639	638
TRRUST	4719	2078	359	337

Target metabolic genes are those reached by at least one regulatory interaction.

In addition, we obtained the most complex *G* matrix and highest computation time with Human1-O2 and Human1-O3 (see Table 2). As it was found in the single-layer analysis, the computation time scales linearly with the number of rows of *G* matrix (Pearson’s correlation = 0.98, *p* value = 3.134e-07).

For each model, we calculated gMCSs until length 5 that block biomass production (Table 2). The effect of the simplified *G* matrix is clearly observed and made it possible the search of gMCSs for the most complex cases. 10091 gMCSs were identified for Human1. All of them were included in our 9 integrated models (Supplementary Fig. 5). Human1-O2 and Human1-O3 increased importantly the number of gMCSs, going from 14060 gMCSs in Human1-O1 to 15104 and 18624 gMCSs in Human1-O2 and Human1-O3, respectively. This is not observed either in Human1-D2 and Human1-D3 or in Human1-T2 and Human1-T3, showing that higher layers do not necessarily incur in an increase of gMCSs, e.g. Human1-D2 has less gMCSs than Human1-D1. Due to the complexity of eGPR rules, a small proportion of gMCSs becomes non-minimal interventions in higher layers and, thus, they are discarded (Supplementary Fig. 6). Again, we observed that the new subset of gMCSs identified strongly depends on the regulatory database employed and the intersection between databases is limited (Supplementary Fig. 5).

**Fig. 2:**
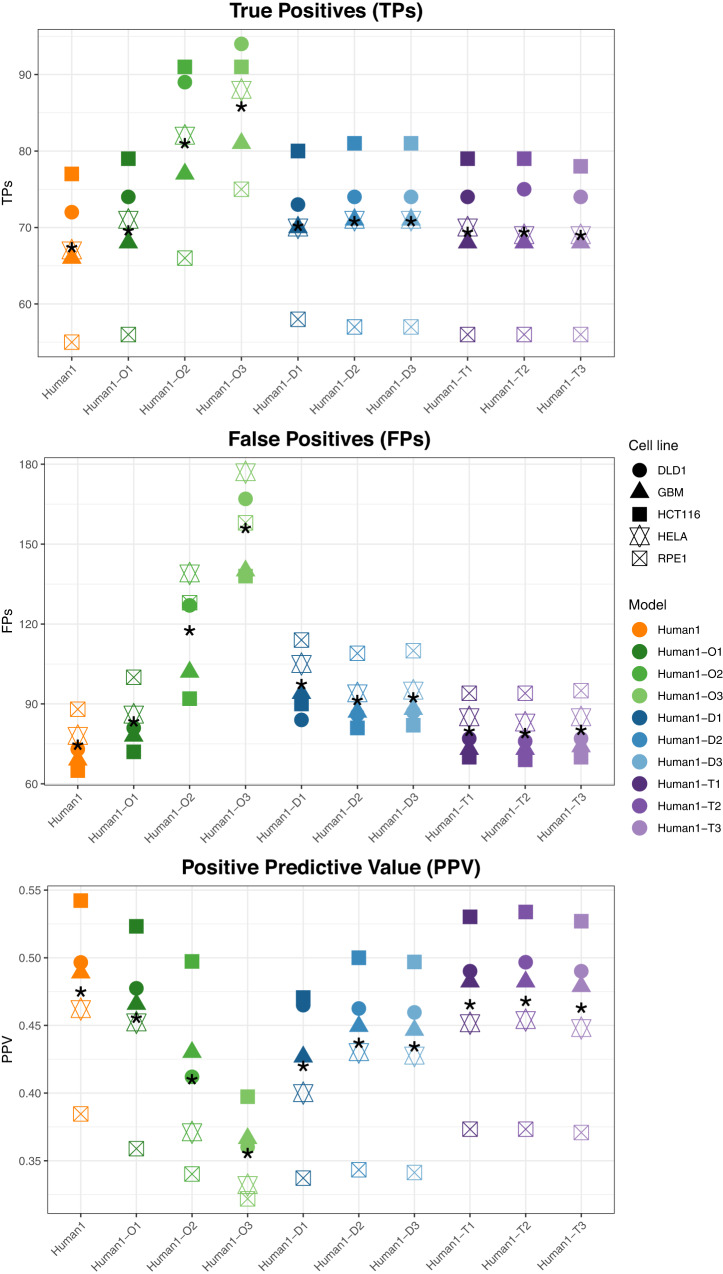
Gene essentiality comparison between Human1 and multiple-layer integrated metabolic and regulatory models. True Positives (TPs), False Positives (FPs), Positive Predictive Value (PPV) and TPs vs PPV arising from our different models (Human1, Human1-O1, Human1-O2, Human1-O3, Human-D1, Human-D2, Human-D3, Human1-T1, Human1-T2, Human1-T3) using the essentiality data presented in Hart2015 as a gold-standard. Asterisk * represents the mean value for the 5 cell lines considered.

We conducted the same gene essentiality analysis shown above for multiple-layer integrated models (Fig. 2). In the case of Human1-O, the number of TPs increased to significantly lower rate than the number of FPs after adding the second and third layer (paired *t* test *p* value ≤ 0.05). For example, 12 TPs and 35 FPs were additionally obtained on average in Human1-O2 with respect to Human1-O1. This substantially decreased average PPV in comparison with Human1-O1, namely from 0.456 in Human1-O1 down to 0.356 in Human1-O3. In the case of Human1-D and Human1-T, the behaviour is completely different, finding slightly more accurate results after adding the second layer: average PPV in Human1-D2 = 0.468 and Huma1-T2 = 0.437. We obtained similar conclusions with DepMap data (Supplementary Fig. 7). Finally, in multiple-regulatory layers, we found a significant positive effect of discarding potential essential genes that involve an adaptation upon their knockout (paired *t* test *p* value ≤ 0.05, Supplementary Fig. 8, Methods section).

## Discussion

The integration of genome-scale metabolic and regulatory models has received considerable attention in the literature. Most algorithms aim to integrate regulatory networks to refine the prediction of metabolic fluxes^16–18^. However, the identification of synthetic lethals from these integrated models has been little explored. Early approaches rely on pathway enumeration, which is not tractable for genome-scale models^24^. Here, using the concept of gMCSs, we present an effective approach to address this issue in large-scale networks.

The search for synthetic lethals in these integrated metabolic and regulatory models poses different challenges. Complex regulatory networks, represented here by Boolean networks, involve negation terms and cycles, which are not present in metabolic GPR rules. In this work, we partially address this problem and adapt our previous gMCS formulation to integrate linear regulatory pathways with negation terms. The consideration of regulatory cycles in our approach is pendant and it will be addressed in future works. This is a non-trivial task since regulatory cycles may lead to complex oscillatory behaviours, depending on initial conditions, and our current eGPR networks require further changes to model them correctly.

Our extended gMCS approach was applied to predict synthetic lethality in human cells. To that end, we integrated the most recent generic metabolic model of human cells, Human1^13^, with Omnipath^14^, Dorothea^19^ and TRRUST^20^. For each regulatory network, we built a different integrated model and effectively enumerated gMCSs. In particular, we present results for these integrated models under single (gMCSs up to length 5) and multiple (gMCSs up to length 5) regulatory layers. Our gMCS approach was effective in all the cases considered, including networks involving more than 4500 genes, which opens the door to incorporate other regulatory layers. The main difficulty in extending our gMCS approach to more complex models and regulation layers lies in the presence of regulatory cycles. In the case of Dorothea and TRRUST, for example, the issue of cycles restricts the application of our approach to models with more than 2 regulatory layers (Table 2). This again highlights the necessity of enhancing our approach by incorporating cyclic Boolean networks.

We assessed the performance of our gMCS approach with gene essentiality data from human cancer cell lines. As shown in Fig. 2, the impact of multiple regulation layers is inconclusive. In the case of Omnipath, the models with multiple regulation layers substantially increased the number of genes and resulting gMCSs, but the accuracy in the essentiality predictions notably decreased. This lack of accuracy may be caused by the fact that Omnipath integrates different sources of information with a different quality of annotation and, thus, annotation errors are propagated along multiple-regulatory layers. In the case of Dorothea and TRRUST, considerably smaller databases than Omnipath, the best performance is found in 2-layer integrated models (Human1-T2 and Human1-D2). This result encourages us to investigate new strategies to incorporate more complex regulatory networks.

We also compared the gene essentiality predictions obtained from our different integrated models. A significant number of new essential genes was predicted from these models. However, the integrated models based on TRRUST returned the most accurate results (Human1-T1 and Human1-T2), but slightly lower than Human1, which has a better proportion of true positives and false positives. This overall decline in precision of our integrated models with respect to Human1 shows the complexity in developing accurate regulatory network models. The definition of more robust regulatory network models is a critical task to reduce the rate of false positives in our integrated models. Moreover, in light of the results with multiple-regulatory layers in Dorothea and TRRUST, we also think that the ability to deal with more complex and cyclic Boolean regulatory networks will impact the accuracy of our integrated models.

We analysed in detail essential genes and synthetic lethals obtained with TRRUST. We found five new essential genes for all cell lines (gMCSs of length (1): E2F1, KLF5, NR1H4, SP1 and SREBF2. We found extensive literature supporting our predictions for E2F1 and KLF5^25,26^. The essentiality of E2F1 and KLF5 in our integrated model is related with the control of key metabolic genes involved in the nucleotide metabolism and fatty acid biosynthesis, respectively. In addition, we found that SP1 is over-expressed in most tumours and an attractive target for cancer cells^27^, and that SREBF2 is essential for tumour growth and initiation in colon cancer^28^. While SP1 is a transcription factor with complex interactions with several metabolic pathways, SREBF2 specifically regulates the transport and biosynthesis of cholesterol. Finally, NR1H4 has been shown to be essential in colon cancer^29^, being specifically associated in our integrated models with the transport of cholesterol and fatty acids.

Regarding the new synthetic lethals and context-specific essential genes obtained with TRRUST, a summary list can be found in Supplementary Table 2. Interestingly, we predicted two essential metabolic genes that were not captured by Human1: PISD and TXN2, which shows the potential of our integrated approach to complement previous predictions. In particular, PISD was predicted essential in HCT116 and HELA cell lines, in line with Bellance and collegues^30^, where they demonstrated that doxorubicin inhibits PISD and induces cell death in HELA cells. Similarly, TXN2 was predicted essential in HELA cells, in agreement with the work presented in Zhang et al.^31^, where they proved that knockdown of TXN2 caused a significant decrease of cell viability in HELA. On the other hand, we predicted the essentiality of CREB1 in all cell lines in Hart2015. CREB1 is a transcription factor that comprises a synthetic lethal with ACACB, a metabolic gene implied in fatty acid biosynthesis and biotin metabolism (Supplementary Fig. 9). ACACB is lowly expressed in all the cell lines, and so, the inhibition of CREB1 leads to cell death. The literature is also supporting of our prediction, since Fang et al.^32^ showed that the downregulation of CREB1 is lethal in HCT116. This synthetic lethal shows again the functional interaction between the metabolic and regulatory layers.

Overall, the proposed gMCS approach opens avenues to predict mechanistically synthetic lethal interactions between metabolic and regulatory genes. The computational and functional (biological) analysis presented here shows that our tool can be robustly used to study the regulation of cancer metabolism and associated dependencies.

## Methods

We present below full details of our mathematical formulation to calculate gMCSs in integrated metabolic and regulatory networks. For completeness, we first introduce our previous gMCS formulation for metabolic networks and illustrate the challenges to be addressed. We also describe the strategy followed to construct integrated models with different acyclic regulatory layers, including specific details of the metabolic and regulatory networks used in the Results section. Finally, we detail the necessary modifications to carry out gene essentiality analysis in integrated networks based on gMCSs and transcriptomic data.

### Enumeration of gMCSs via mixed-integer linear programming

Assume we have a metabolic network involving *m* metabolites and *n* reactions. This is typically represented with the stoichiometry matrix *S*, where each column represents a different reaction and each row a single metabolite. Reaction products and substrates have positive and negative coefficients, respectively. The flux vector *r* denotes the activity of the reactions. Here, reversible reactions were split into two irreversible steps and, therefore, reaction fluxes are non-negative (Eq. (1)).

1 $$r\ge 0$$

The application of the mass balance equation under steady state leads to Eq. (2), where the sum of fluxes that produce a certain metabolite is equal to the sum of fluxes that consume it.

2 $$S{{\cdot }}\,r=0$$

Our objective is to block a given metabolic task making use of the least number of gene knockouts. The metabolic task to disrupt can be represented as in Eq. (3):

3 $${t}^{T}{{\cdot }}r\ge {r}^{* },$$

being *t* a null vector with a 1 in the position of the reactions involved in the metabolic task to target and r* a positive constant.

**Fig. 3:**
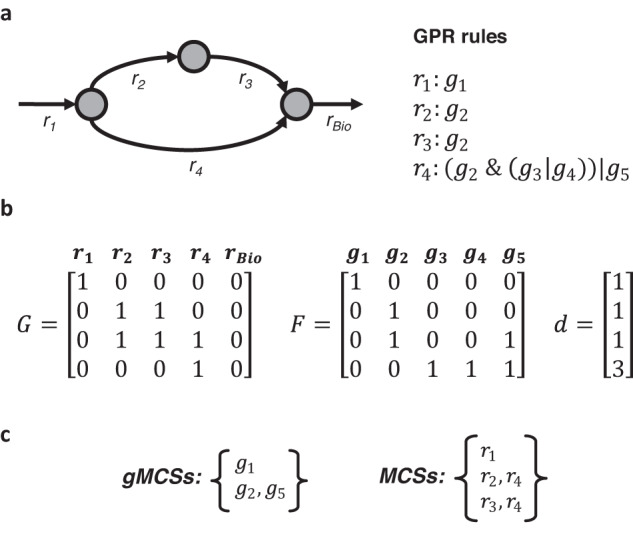
Illustration of gMCSs and MCSs. **a** Example metabolic network and GPR rules. It involves 5 reactions (*r*_*1*_, *r*_*2*_, *r*_*3*_, *r*_*4*_, *r*_*bio*_) and 5 genes (*g*_*1*_, *g*_*2*_, *g*_*3*_, *g*_*4*_, *g*_*5*_); **b** Matrices of gene knockout constraints, *G* and *F*, and net contribution of each row in *G* in terms of gene knockouts, *d*. For example, the second row in *G* is dependent on the third row in *G* and, thus, *d*_*3*_ = 2–1 = 1; **c** Resulting set of gMCSs and MCSs to block the biomass reaction (*r*_*bio*_).

In order to calculate gMCSs, *i.e*. minimal subsets of gene knockouts that disrupt an essential metabolic task, we need to define the possible gene knockout constraints, which take the following form:

4 $$G{{\cdot }}r\le 0,$$

where the binary *G* matrix, of dimensions *l*x*n*, defines for each row *i* the set of blocked reactions, *G(i)* *=* *{k|G*_*ik*_ = *1}*, arising from the knockout of an irreducible subset of genes. The subset of genes associated with each row in *G* is interrelated and their simultaneous knockout is required to delete at least one of the reactions in the metabolic network. This information is stored in the binary matrix *F* of dimensions *l*x*g*, which defines the subset of gene deletions involved in each row *i* in *G*, *F(i)* *=* *{p|F*_*ip*_ = *1}*. In other words, the deletion of genes in *F(i)* leads to the disruption of reactions in *G(i)*. An example metabolic network, including gene-protein-reaction (GPR) rules, can be found in Fig. 3a. For illustration, Fig. 3b show its associated *G* and *F* matrices, where, according to their second row, the knockout of gene 2 (*g*_*2*_) leads to the blockage of reaction 2 and 3 (*r*_*2*_, *r*_*3*_).

From the infeasible primal problem defined by Eqs. (1–4), we formulate the unbounded dual problem and minimise the number of gene knockouts to block the target metabolic task with the following mathematical model:

5 $${minimize}\,\mathop{\sum }\limits_{i=1}^{i=l}{{d}_{i}\cdot z}_{i}$$

*s.t*.

6 $$N{{\cdot }}\left(\begin{array}{c}u\\ v\\ w\end{array}\right)=\left[\begin{array}{ccc}{S}^{T} & {G}^{T} & -t\end{array}\right]{{\cdot }}\left(\begin{array}{c}u\\ v\\ w\end{array}\right)\ge 0$$

7 $$\alpha z\le v\le {Mz}$$

8 $${r}^{* }{{\cdot }}w\le -c,c \,>\, 0$$

9 $${z}_{\delta }\ge {z}_{\beta }\,\forall \left(\delta ,\beta \right){\rm{|}}F\left(\beta \right)\supset F\left(\delta \right)$$

10 $$\mathop{\sum }\limits_{i=1}^{i=l}{z}_{i}^{j}{z}_{i}\le \mathop{\sum }\limits_{i=1}^{i=l}{z}_{i}^{j}-1$$

11 $$v\ge 0,w\ge 0$$

12 $$u\in {R}^{m},v\in {R}^{l},w\in R,z\in {B}^{l}$$

where *u*, *v,* and *w* are dual variables associated with the mass balance equation, gene knockout constraints, and the target metabolic task equation, respectively; *z* are binary variables linked to *v* through Eq. (7), namely *z* = 0 ↔ *v* = 0, *z* = 1 ↔ *v* > 0. Note here that *α* and *M* are small and large positive constants, respectively. Equation (8) forces *w* to be non-zero, which makes the target metabolic task equation part of the infeasible primal problem. Equation (9) considers the dependencies between dual variables *v* that may lead to non-minimal solutions, as it is described in Apaolaza et al.^8^. In addition, *d* is a known vector storing the number of gene deletions exclusively provided by its associated dual variable *v* and not by its dependent dual variables (see Fig. 3b for illustration). Dependencies between dual variables can be easily obtained from *F* matrix. Finally, Eq. (10) allows us to eliminate previously obtained solutions (*z*^*j*^) from the solution space and identify new gMCSs.

In summary, the mixed-integer linear programme defined by Eqs. (5)–(12) (MILP1) allows us to enumerate gMCSs in increasing order of gene knockouts. Figure 3c shows the resulting set of gMCSs for the example network considered. Note here that a similar approach can be built for Minimal Cut Sets (MCSs), which involves reaction knockouts instead of gene knockouts, as developed in different works^33^ (Fig. 3c). In particular, for the computation of MCSs, the matrix *G* in Eq. (6) becomes the identity matrix (if all reactions are irreversible) and, thus, dependency constraints in Eq. (9) can be neglected.

### Calculation of G matrix in metabolic networks

MILP1 requires as input data different matrices: *S*, *G*, *F* and *t*. The construction of *G* and *F* matrices is not a trivial task, as demonstrated in Apaolaza et al.^8^ where we presented an efficient algorithm for their computation in complex metabolic networks. This technical improvement has allowed us to enumerate thousands of gMCSs in genome-scale metabolic networks in human cells^11^.

**Fig. 4:**
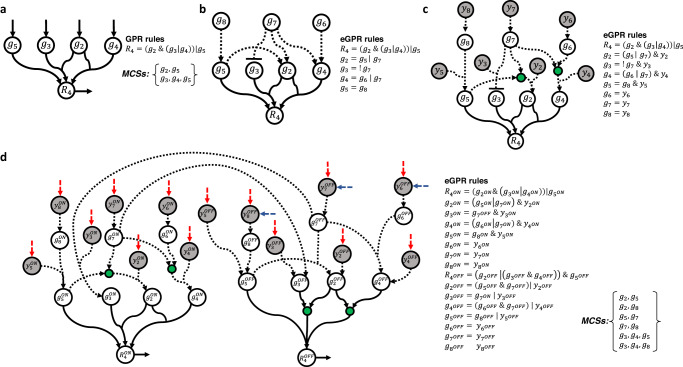
Illustration of extended GPR networks. **a** Resulting GPR network for reaction 4 (*r*_*4*_) in Fig. 3, its associated GPR rules and output MCSs; **b** GPR rules including the regulation of metabolic genes involved in (*r*_*4*_) using Boolean equations. Three new genes are incorporated into eGPR rules: *g*_*6*_, *g*_*7*_, *g*_*8*_; **c** Addition of auxiliary nodes *y* representing gene knockouts in Boolean equations; **d** resulting extended GPR (eGPR) network after dividing each node into two different ON/OFF nodes and including input and output exchange reactions. Regulatory interactions are represented through arcs in dashed lines.

Our *G* matrix construction algorithm involves 2 stages: (i) calculation of irreducible subsets of gene knockouts that block each reaction separately using GPR rules; (ii) integration of these irreducible subsets for the definition of *G* and *F* matrices. The first stage is the most challenging part, but it could be elegantly solved by transforming GPR rules into artificial reaction networks, called here GPR networks, and apply the MCS approach to block the target reaction^8^ only considering the deletion of exchange reactions. Figure 4a shows the GPR rule for reaction 4 (*r*_*4*_) present in the example in Fig. 3, the associated GPR network and the 2 resulting MCSs. This strategy could be followed because GPR rules define Boolean networks that do not involve (i) negation (inhibition) terms and (ii) cycles that could lead to oscillatory behaviour, as it is typically found in complex regulatory networks.

Here, we extend our computational approach to calculate gMCSs in metabolic networks that integrate linear (acyclic) regulatory pathways. In particular, we amend the *G* matrix construction algorithm to deal with the resulting acyclic Boolean networks that control metabolic reactions. The inclusion of inhibitory interactions (negation terms) in regulatory pathways requires the redefinition of our previous GPR networks and the algorithm to calculate MCSs. Figure 4b shows an example reaction that includes the regulatory information for the genes implied in its associated GPR rule. We describe below how these extended GPR (eGPR) rules are transformed into reaction networks, referred to now as extended GPR (eGPR) networks, and how the MCS approach is applied to them.

### Calculation of G matrix in integrated metabolic and regulatory networks

1. Construction of eGPR networks. For the sake of clarity, for each target reaction *k*, denoted *R*_k_, we define *B*(*k*) as the subset of genes implied in its associated eGPR rules. Each of these genes, denoted *g*_*i*_ (*i* = 1,…, |*B*(*k*)|), are interconnected through their corresponding Boolean equations. We denote *L*(*k*) the subset of those nodes without Boolean equations (in Fig. 4b, we have *g*_6_, *g*_7_ and *g*_8_). Nodes in *L*(*k*) represent input genes for the resulting Boolean network and can freely take 0/1 values. In order to build the eGPR network for each reaction, we follow 5 different steps:

i. The Boolean equation for each gene in *B*(*k*) is first updated with a necessary auxiliary node *y*_*i*_ (*i* = 1,…, | *B*(*k*)|), which allows us to consider the effect of gene knockouts without affecting the network upstream. The resulting Boolean network and updated eGPR rules can be found in Fig. 4c. Note here that we introduce intermediate nodes (shown in green) to consider OR rules.
ii. Nodes from the Boolean network in the previous step are split into ON and OFF nodes, namely ${y}_{i}^{{ON}}$, ${y}_{i}^{{OFF}}$, ${g}_{i}^{{ON}}$, ${g}_{i}^{{OFF}}$, ${R}_{k}^{{ON}}$, ${R}_{k}^{{OFF}}$ and, following the De Morgan’s laws, eGPR rules are updated. This strategy duplicates the number of nodes and interactions but negation terms disappear from the Boolean equations, which make it possible to model them as a reaction network. The resulting network is shown in Fig. 4d.
iii. Addition of an input exchange reaction for nodes with no input arcs, namely ${y}_{i}^{{ON}}$ and ${y}_{i}^{{OFF}}$. The removal of these exchange reactions represents the knockout/activation of the genes involved in our reaction network. This set of input exchange reactions is denoted *Y*(*k*). They are coloured red in Fig. 4d.
iv. Addition of an input exchange reaction for ${g}_{i}^{{OFF}}$ nodes such that $i\in$*L*(*k*). In general, we can reach ${g}_{i}^{{OFF}}$ nodes from different pathways but, in the case of input genes *L*(*k*), ${g}_{i}^{{OFF}}$ can be freely active (depending on the initial conditions). They are coloured blue in Fig. 4d.
v. Addition of an output exchange reaction for ${R}_{k}^{{ON}}$ and ${R}_{k}^{{OFF}}$, which are denoted, respectively, ${r}_{{k}^{{ON}}}$ and ${r}_{{k}^{{OFF}}}$ (see Fig. 4d).

2. Calculation of MCSs in eGPR networks. eGPR networks can be modelled as a reaction system that satisfies irreversibility constraints and the mass balance equation:

13 $${r}^{k}\ge 0$$

14 $${S}^{k}{{\cdot }}{r}^{k}=0,$$

where *r*^*k*^ denotes the flux through the artificial reactions involved in the eGPR network for the target reaction *k* and *S*^*k*^ its associated stoichiometry matrix of dimensions *m*^*k*^x*n*^*k*^.

In order to calculate MCSs that blocks the target reaction ${R}_{k}^{{ON}}$, we can adapt Eq. (3) to force flux through this reaction and Eq. (4) to define the knockout space for the input exchange reactions in *Y*(*k*)*:*

15 $${t}_{{R}_{k}^{{ON}}}^{T}{{\cdot }}{r}^{k}\ge {r}^{* }$$

16 $${r}_{i}^{k}\le 0\,\forall i\in Y(k),$$

where${t}_{{R}_{k}^{{ON}}}^{T}$ is a null vector with a 1 in the position of the target reaction ${R}_{k}^{{ON}}$. Note here that in Eq. (16) we only include input exchange reactions in *Y*(*k*) because they represent the decision as to whether (or not) a gene is knocked out. The knockout of ${y}_{i}^{{ON}}$ and ${y}_{i}^{{OFF}}$ nodes are not independent, but they are coordinated in the dual problem that is presented below.

The dual problem of this infeasible primal problem, Eqs. (13)–(16), takes a similar form than the one presented in Eqs. (5)–(12):

17 $${\rm{minimize}}\mathop{\sum} \limits_{i=1}^{i={\rm{|}}B(k){\rm{|}}}{z}_{{y}_{i}^{{ON}}}^{k}$$

*s.t*.

18 $$\left[{{S}^{k}}^{T}I-{t}_{{R}_{k}^{{ON}}}\right]\left(\begin{array}{c}{u}^{k}\\ {v}^{k}\\ {w}^{k}\end{array}\right)\ge 0$$

19 $${v}^{k}\ge 0,{w}^{k}\ge 0$$

20 $${u}^{k}\in {R}^{{m}^{k}},{v}^{k}\in {R}^{\left|Y\left(k\right)\right|},{w}^{k}\in R,{z}^{k}\in {B}^{{l}^{k}}$$

21 $${r}^{* }{w}^{k}\le -c$$

22 $$\alpha {z}^{k}\le {v}^{k}\le M{z}^{k}$$

23 $${z}_{{y}_{i}^{{ON}}}^{k}+{z}_{{y}_{i}^{{OFF}}}^{k}=1,i=1,\ldots ,\left|B\left(k\right)\right|$$

24 $$\mathop{\sum}\limits_{i=1}^{i={\rm{|}}B(k){\rm{|}}}{z}_{{y}_{i}^{{ON}}}^{k}\ge 1$$

25 $$\mathop{\sum}\limits_{i=1}^{i={\rm{|}}B(k){\rm{|}}}{{z}_{{y}_{i}^{{ON}}}^{k}}^{j}{z}_{{y}_{i}^{{ON}}}^{k}\le \mathop{\sum}\limits_{i=1}^{i={\rm{|}}B(k){\rm{|}}}{{z}_{{y}_{i}^{{ON}}}^{k}}^{j}-1$$

However, Eqs. (17)–(25), called MILP2, differ from MILP1 in the following points:

(i) the knockout space only considers input exchange reactions associated with ${y}_{i}^{{ON}}$ and ${y}_{i}^{{OFF}}$, which allow us to decide which gene *i* is knocked out (${r}_{{y}_{i}^{{ON}}}\le 0$) or not (${r}_{{y}_{i}^{{OFF}}}\le 0$) to block the target reaction;

(ii) Eq. (23) forces that for each gene *i* exactly one these two constraints: ${r}_{{y}_{i}^{{ON}}}\le 0$ and ${r}_{{y}_{i}^{{OFF}}}\le 0$ takes part in the infeasible primal problem. This constraint is specific of MILP2 and it is due to the inherent coupling between ${y}_{i}^{{ON}}$ and ${y}_{i}^{{OFF}}$ nodes. This constraint establishes that if a gene is knocked out, *i.e*. ${r}_{{y}_{i}^{{ON}}}\le 0$, then ${r}_{{y}_{i}^{{OFF}}}$ cannot be forced to be zero and vice versa;

(iii) the objective function, Eq. (17), minimises the number of knockouts of input exchange reactions associated with ${y}_{i}^{{ON}}$, since they represent gene knockouts (${y}_{i}^{{ON}}\le 0$). The same logic applies to the solution elimination constraint in Eq. (25);

(iv) we force the optimal solution to involve at least one gene knockout in Eq. (24).

MILP2, Eqs. (17)–(25), allows us to enumerate MCSs for eGPR networks. Figure 4d shows the resulting MCSs for the eGPR network of reaction 4 in Fig. 3.

**Fig. 5:**
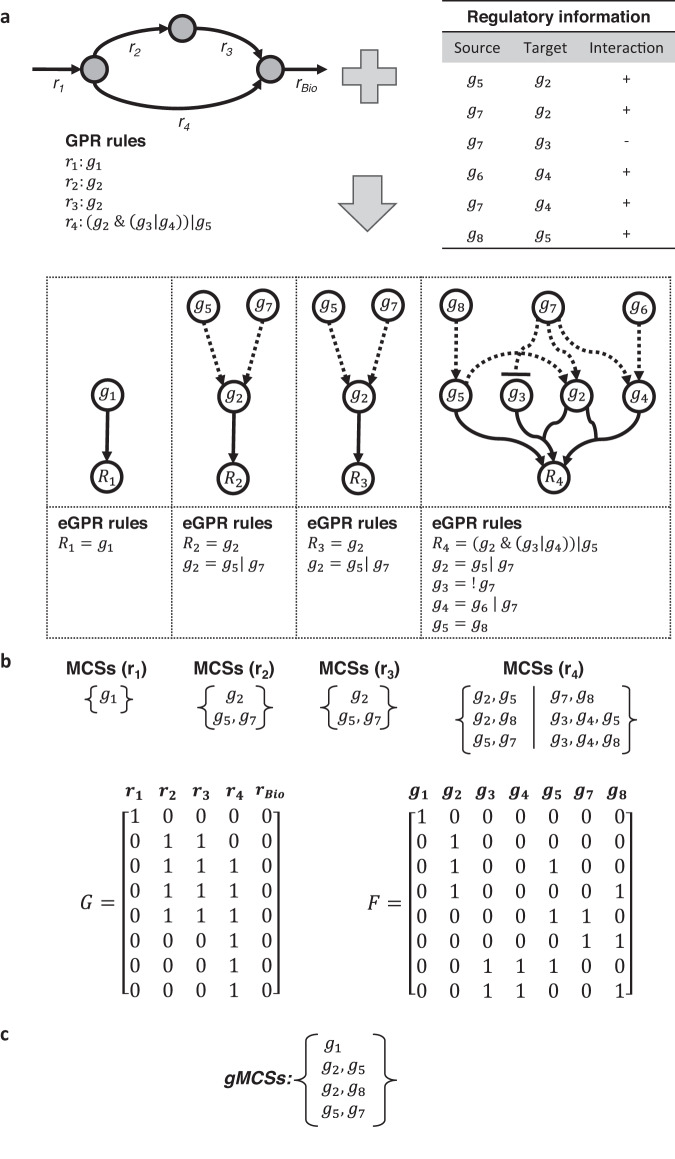
Illustration of gMCSs in integrated metabolic and regulatory models. **a** Example integrated metabolic and regulatory model that extends the metabolic network in Fig. 3. **b** Resulting MCSs for each target reaction after applying MILP2 to its associated eGPR network. In addition, *G* and *F* matrices are provided. **c** Resulting gMCSs to block the biomass reaction (*r*_*bio*_) for this toy example integrated network.

3. Calculation of G matrix. Using as input data the GPR rules and regulation available for a specific metabolic network, MILP2 is applied to each different reaction. For illustration, Fig. 5a shows the example metabolic network in Fig. 3a, but additionally including the regulation for some of the metabolic genes involved (eGPR rules). Figure 5b shows the resulting MCSs for each target reaction after applying MILP2 to its associated eGPR network. MCSs for different reactions are then integrated in order to build G and F matrix (see Fig. 5b). We have developed a MATLAB function for building the G matrix in integrated metabolic and regulatory models, called ‘*buildGmatrix_iMRmodel*’, which is in the COBRA toolbox^34^. Note here that as the size of G matrix increases with the addition regulatory interactions, we have conducted several improvements in this function, reducing up to 3 times the computation time with respect our previous implementation. Moreover, we give the possibility to remove rows in *G* matrix involving a higher number of genes than a specified length value. For example, if we aim to search for gMCSs up to length 5, we can delete rows in *G* involving more than 5 genes. This simplified *G* matrix substantially reduces the computational burden of the search process.

Once the *G* matrix has been obtained, the list of gMCSs can be calculated using the function ‘*calculateGeneMCS’*, also presented in Apaolaza et al.^8^, which makes use of MILP1. The resulting gMCSs for our toy example can be found in Fig. 5c.

### Definition of regulation layers in metabolic models

In order to define the regulation layer of the metabolic network under study, we first find, using different databases (see next sub-section), signed interactions for each metabolic gene involved in GPR rules. Then, we create a new Boolean equation that integrates the identified interactions for each metabolic gene using ‘OR’ operators, as observed in Fig. 5a, leading to eGPR rules.

As noted above, the methodology developed in this work (MILP2) is not able to deal with cyclic behaviours that are common in Boolean networks. For that reason, at the time of adding a regulatory layer, we must check that there are no cycles in the resulting eGPR network. This is done by solving the following linear programming problem (LP1) for each reaction *R*_*k*_:

26 $${\rm{minimize}}\mathop{\sum}\limits_{i=1}^{i={n}^{k}}{r}_{i}^{k}$$

*s.t*.

27 $$\mathop{\sum}\limits_{i=1}^{i={n}^{k}}{r}_{i}^{k}\ge 1$$

28 $${S}^{k}{{\cdot }}{r}^{k}=0$$

29 $${r}_{i}^{k}=0,i\in {E}^{k}$$

30 $${r}^{k}\ge 0,$$

where ${E}^{k}$ is the subset of input and output exchanges in the eGPR network for reaction *R*_*k*_.

If we delete input and output exchanges fluxes with Eq. (29), LP1 is only feasible in the case we have cycles in the eGPR network, otherwise the solution is infeasible. Once it is tested that the eGPR network does not present cycles (LP1 is infeasible), the regulatory layer is added to the model. Note here that adding a layer involves including more genes to the model which can be regulated by other genes. Therefore, we can search for all the regulatory interactions related to the genes added in the previous layer and insert new genes to the network as explained above. Then, the absence of cycles is checked and the layer is added. This process can be repeated as many times as layers are desired to be added to the model. Supplementary Fig. 10 shows the toy example in Fig. 3 with one, two and three regulation layers.

### Regulatory and metabolic networks of human cells

To assess our extended approach, we employed the protein-protein interaction network of Omnipath^23^ (accessed online 2023-04-03) (OmnipathR, v.3.0.4), the gene regulatory network of signed transcription factors Dorothea^19^ (dorothea, v.1.7.2) and the manually curated database of human transcriptional regulatory networks TRRUST^20^. The main characteristics of each regulatory network are shown in Table 3.

To avoid unnecessary noise in our integrated models, we filtered the interactions of each database without a defined sign (activation or inhibition). Surprisingly, we found a limited overlap between different regulatory networks in terms of genes and interactions (Supplementary Fig. 11).

Regarding the metabolic model, we used the most recent genome-scale metabolic network of human cells: Human1 (v1.14.0)^13,22^, obtained from https://github.com/SysBioChalmers/Human-GEM. Human1 involves 8363 metabolites, 2920 genes and 13024 reactions. Although this model defines 56 essential metabolic tasks, for simplicity, we have focused on the task of biomass production in this work.

Human1 makes use of Ham’s medium to produce biomass. Therefore, the flux through the input exchange reactions of metabolites not involved in Ham’s medium, as defined in Human1 for biomass production, was set to zero. Then, the model was simplified with the function *simplifyModel* of RAVEN^35^, deleting reactions that are constrained to zero flux. After this simplification, Human1 is reduced to 6830 metabolites, 2419 genes and 11573 reactions.

### Gene essentiality analysis

We classify a gene as potentially essential in a particular sample if it is the unique highly expressed gene in at least in one gMCS and the rest of the genes of that gMCS are lowly expressed, as done in Valcárcel et al.^11^. For the definition of highly and lowly expressed genes for each sample, we applied the *gmcsTH5* threshold presented in that work. In brief, the *gmcsTH5* thresholding technique assumes that each gMCS should have at least one highly expressed gene to guarantee the feasibility of the target metabolic task, in our case the biomass reaction. Under this assumption, an empirical probability function of the expression of highly expressed genes is obtained for each sample, namely by extracting for every gMCS the gene with maximum expression (repeats are avoided). For each sample, *gmcsTH5* refers to the 5% quantile expression threshold of this probability function. Thus, highly expressed genes are those with a higher expression than *gmcsTH5*. For consistency, *gmcsTH5* was derived for each sample using the gMCSs calculated for Human1 and applied to the rest of the integrated models.

Once we have identified potential essential genes in a sample, we need to ensure that, when they are knocked out, the rest of the genes participating in the gMCSs of interest do not become active by means of an adaptation mechanism. In contrast with GPR rules in metabolic networks, the presence of negation terms in eGPR rules may support this adaptation upon gene knockout (see an example network in Supplementary Fig. 12).

To assess the presence of adaptation pathways, we integrate all eGPR rules and transform their Boolean equations into linear constraints with binary variables *x*, similar to the work presented in Shlomi et al.^36^. Note here that we include auxiliary nodes *y* in eGPR rules to model gene knockouts, as done in Fig. 4c. Then, we force the knockout of a potential essential gene *T* and minimise the number of genes involved in its associated ‘explaining gMCS’, *P*_*T*_. We define here an ‘explaining gMCS’ as one that explains the potential essentiality of a target gene *T*. This problem can be solved via integer linear programming (ILP1):

31 $${\rm{minimize}}\sum _{j\in {P}_{T}}{x}_{j}$$

*s.t*.

32 $$\alpha \le {A\cdot x}\le \beta$$

33 $${x}_{{Y}_{T}}=0$$

34 $$x:\left\{\mathrm{0,1}\right\}$$

If the objective value for ILP1 is zero, gene essentiality remains. However, if the objective value is greater than zero, we have adaptation pathways and the essentiality of target gene *T* is discarded. For simplicity, we calculated the list of single gene knockouts and associated gMCSs that present an adaptation pathway. If a potential essential gene *T* and its ‘explaining gMCS’ are present in this list, the essentiality of *T* is directly discarded.

### Implementation

For the different studies conducted in the Results section, we used the University of Navarra’s computing cluster, limiting to 8 cores and 8 GB of RAM. A time limit of 5 min was set for each solution derived from the function ‘*CalculateGeneMCS’*. MATLAB and The COBRA toolbox was used to implement the function ‘*buildGmatrix_iMRmodel*’, with help of IBM Ilog Cplex for the underlying MILP model.

### Reporting summary

Further information on research design is available in the Nature Research Reporting Summary linked to this article.

## Supplementary information

Supplementary Material

Reporting Summary

## Data Availability

The authors confirm that the data supporting the findings of this study are available within the article and its supplementary material.

## Code availability

The full code to generate the results and figures presented in the article, as well as the detailed list of gMCSs from all model combinations, are now available in https://github.com/PlanesLab/iMR_gmcs_paper_scripts.
