# Supplementary material for: Synthetic lethality in large-scale integrated metabolic and regulatory network models of human cells
Source: NPJ Syst Biol Appl. 2023 Jul 15;9:32. doi: 10.1038/s41540-023-00296-3 (PMC10349875; doi:10.1038/s41540-023-00296-3)
Supplement: Supplementary file 1 — Supplementary Material [file 41540_2023_296_MOESM1_ESM.pdf]

Supplementary Figures and Tables: Synthetic lethality in large-scale integrated metabolic and regulatory network models of human cells

Naroa Barrena[1], Luis V. Valcárcel[1,2,3], Danel Olaverri-Mendizabal[1], Iñigo Apaolaza[1,2,3], Francisco J. Planes[1,2,3,*]

[1]University of Navarra, Tecnun School of Engineering, Manuel de Lardizábal 13, 20018 San Sebastián, Spain.

[2]University of Navarra, Biomedical Engineering Center, Campus Universitario 31009 Pamplona, Navarra, Spain.

[3]University of Navarra, Instituto de Ciencia de los Datos e Inteligencia Artificial (DATAI), Campus Universitario, 31080, Pamplona, Spain

[*] *Corresponding author:* fplanes@tecnun.es

Supplementary Figures

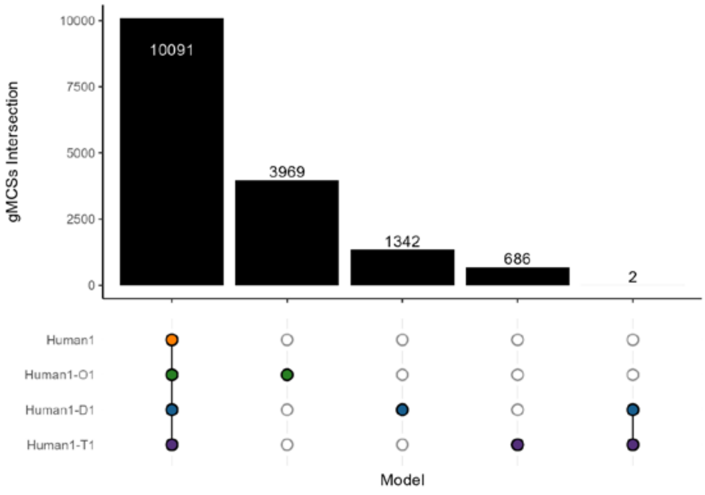

Supplementary Figure 1. Analysis of gMCSs obtained from single-layer integrated metabolic and regulatory models. UpSet plot representing the intersections of gMCSs until length 5 calculated from different integrated models: Human1, Human1-O1, Human-D1, Human1-T1. Abbreviations: '*Human1-O1*': integrated model with Human1 and Omnipath with one regulatory layer; '*Human1-D1*': integrated model with Human1 and Dorothea with one regulatory layer; '*Human1-T1*': integrated model with Human1 and TRRUST with one regulatory layer.

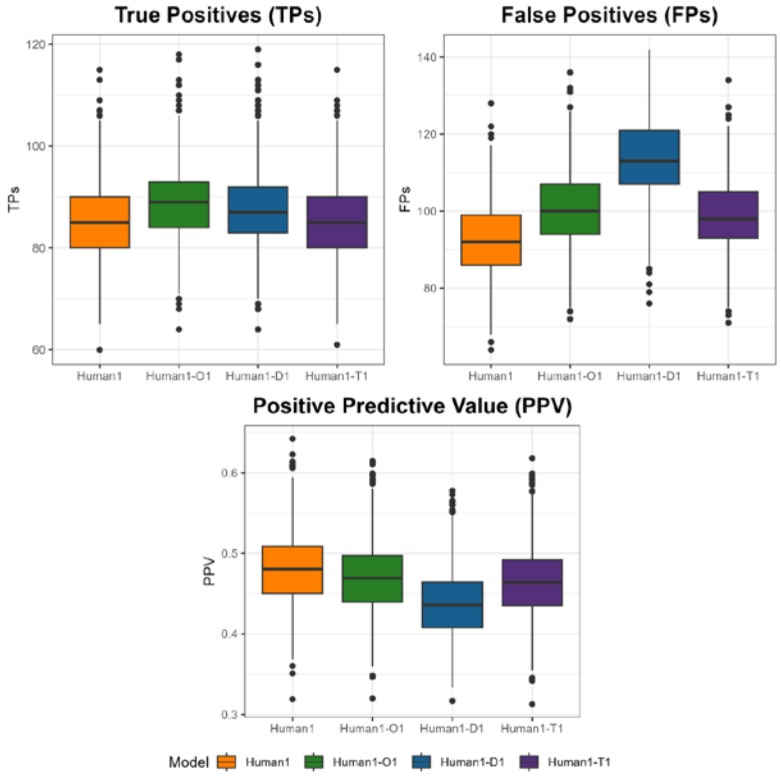

Supplementary Figure 2. Gene essentiality comparison between Human1 and single-layer integrated metabolic and regulatory models using data from DepMap. True Positives (TPs), False Positives (FPs) and Positive Predictive Value (PPV) arising from our different models (Human1, Human1-O1, Human-D1, Human1-T1) using the essentiality data presented in DepMap. In the boxplots, the center line represents the median, the bounds of the box correspond to the 25th and 75th percentiles, the whiskers indicate the interquartile range by 1.5 times and the circles are the outliers.

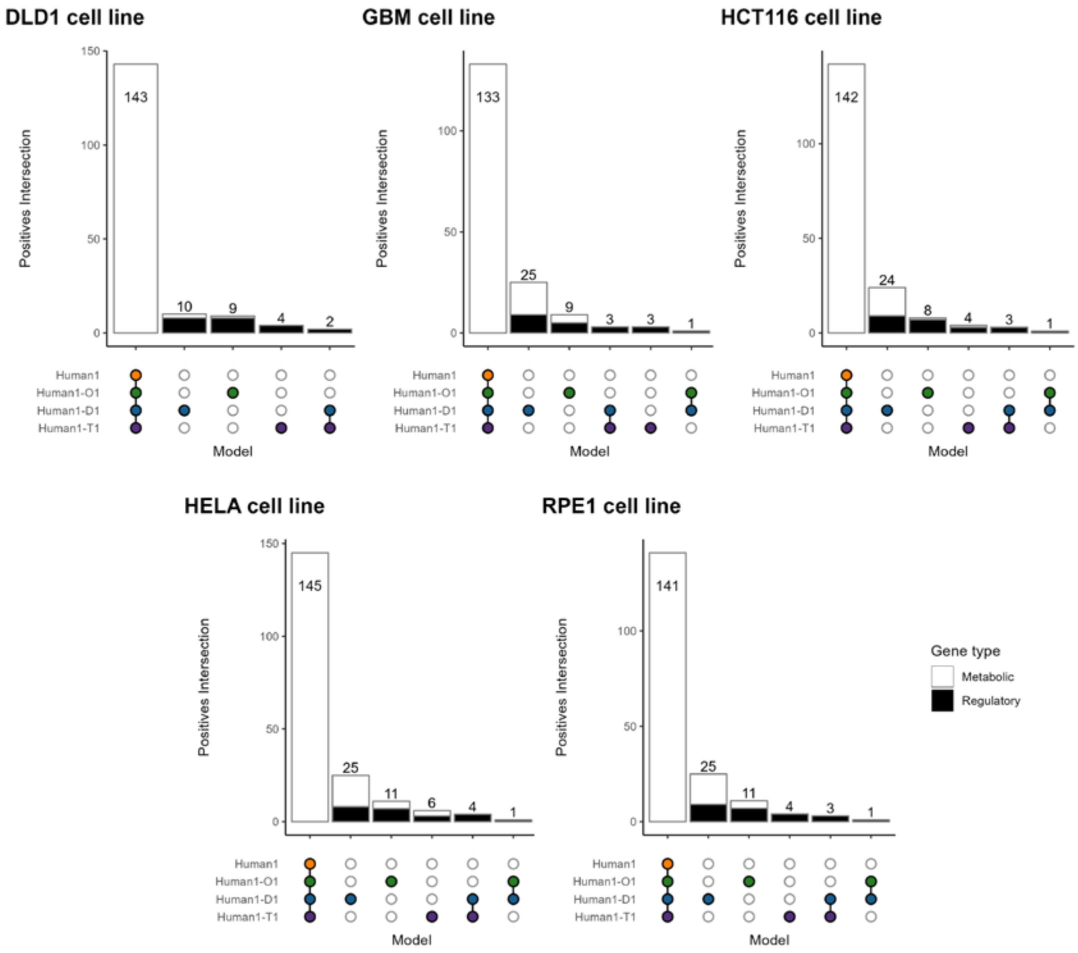

Supplementary Figure 3. Predicted number of essential genes in each cell line of Hart2015 with Human1 and single-layer integrated metabolic and regulatory models. UpSet plots representing the intersection of the predicted essential genes in Hart2015 cell lines (DLD1, GBM, HCT116, HELA, RPE1) derived from the gMCSs obtained from the different models analyzed: Human1, Human1-O1, Human-D1, Human1-T1.

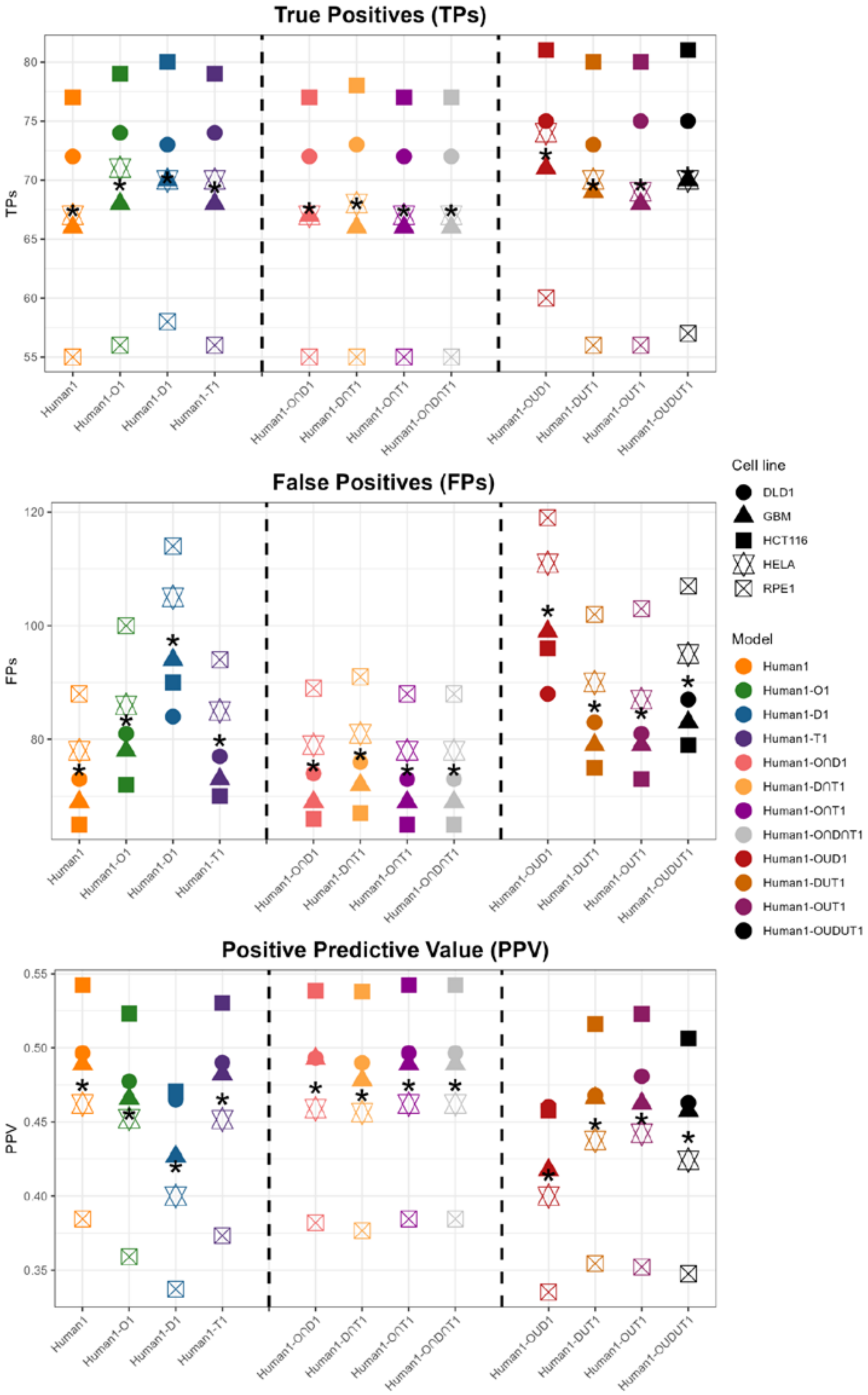

Supplementary Figure 4. Effect of the combination of different regulatory databases in the gene essentiality comparison with Hart2015. True Positives (TPs), False Positives (FPs) and Positive Predictive Value (PPV) arisings from our different single-layer integrated metabolic and regulatory models considered: Human1, Human-O1, Human-D1, Human1-T1, Human1-O∩D1 (Human1 + the intersection of Omnipath and Dorothea), Human1-D∩T1 (Human1 + the intersection of Dorothea and TRRUST), Human1-O∩T1 (Human1 + the intersection of Omnipath and TRRUST), Human1-O∩D∩T1 (Human1 + the intersection of Omnipath, Dorothea and TRRUST), Human1-OUD1 (Human1 + the union of Omnipath and Dorothea), Human1-DUT1 (Human1 + the union of Dorothea and TRRUST), Human1-OUT1 (Human1 + the union of Omnipath and TRRUST), Human1-OUDUT1 (Human1 + the union of Omnipath, Dorothea and TRRUST). Asterisk * represents the mean value for the 5 cell lines considered.

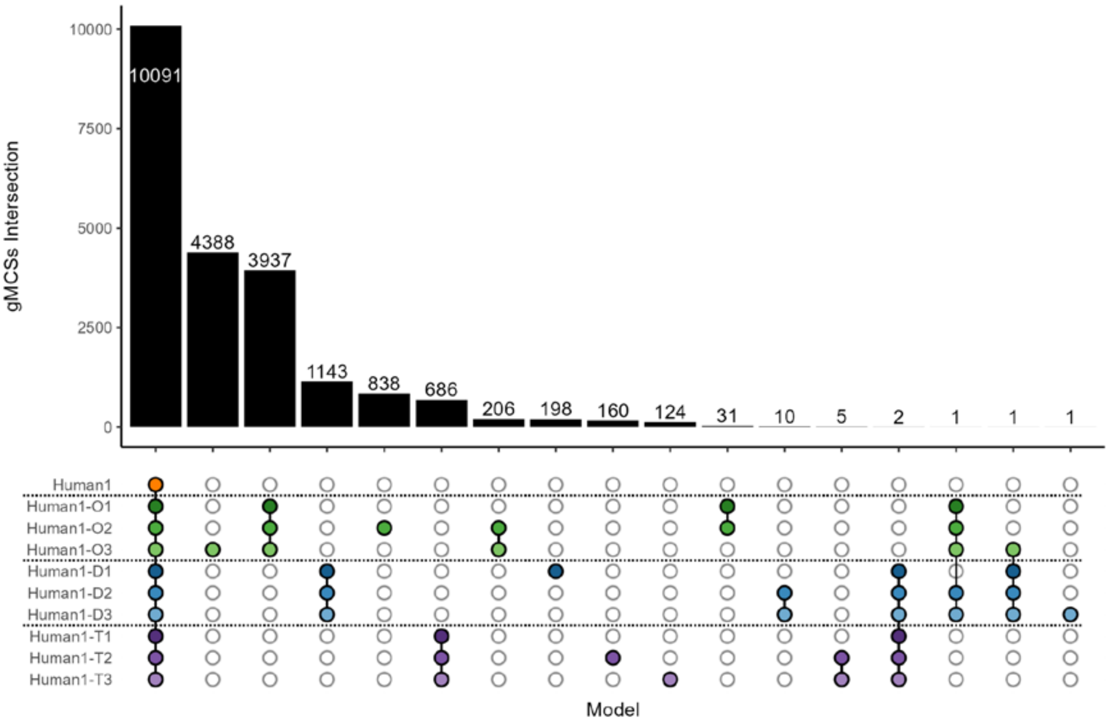

Supplementary Figure 5. Analysis of gMCSs obtained from multiple-layer integrated metabolic and regulatory models. Upsetplot representing the intersection of the gMCSs until length 5 calculated in our different models: Human1, Human1-O1, Human1-O2, Human1-O3, Human-D1, Human-D2, Human-D3, Human1-T1, Human1-T2, Human1-T3. Abbreviations: 'Human1-O1': integrated model with Human1 and Omnipath with one regulatory layer; 'Human1-O2': integrated model with Human1 and Omnipath with two regulatory layers; 'Human1-O3': integrated model with Human1 and Omnipath with three regulatory layers; 'Human1-D1': integrated model with Human1 and Dorothea with one regulatory layer; 'Human1-D2': integrated model with Human1 and Dorothea with two regulatory layers; 'Human1-D3': integrated model with Human1 and Dorothea with three regulatory layers; 'Human1-T1': integrated model with Human1 plus TRRUST and one regulatory layer; 'Human1-T2': integrated model with Human1 plus TRRUST and two regulatory layers; 'Human1-T3': integrated model with Human1 and TRRUST with three regulatory layers.

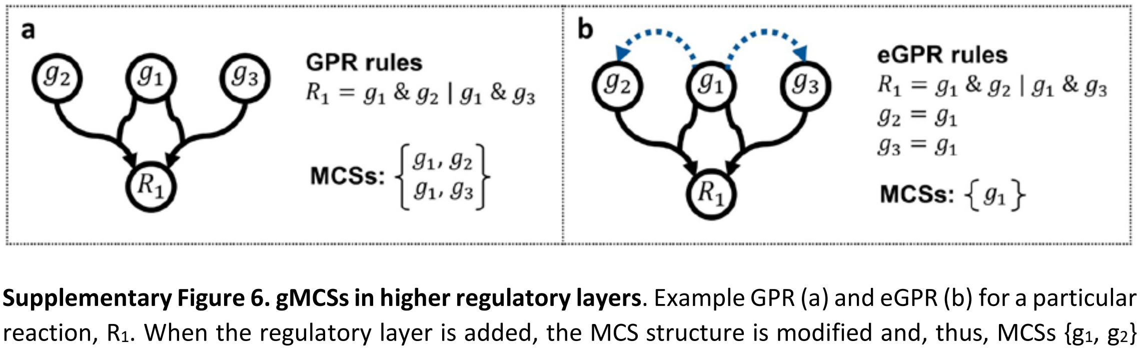

Supplementary Figure 6. gMCSs in higher regulatory layers. Example GPR (a) and eGPR (b) for a particular reaction, R_1. When the regulatory layer is added, the MCS structure is modified and, thus, MCSs $\{g_1, g_2\}$ and $\{g_1, g_3\}$ are substituted by $\{g_1\}$. In this case, the number of gMCSs decreases when a regulatory layer is included in the model.

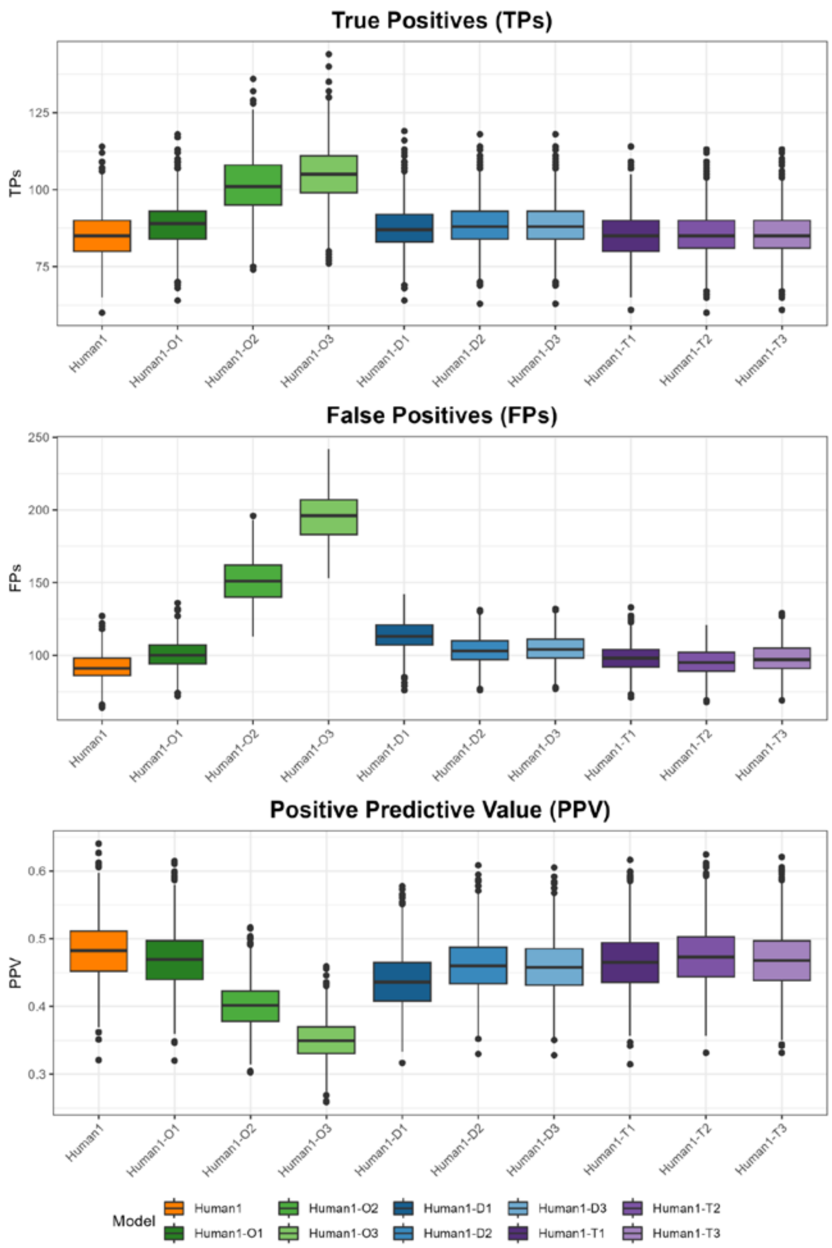

Supplementary Figure 7. Gene essentiality comparison between Human1 and multiple-layer integrated metabolic and regulatory models using data from DepMap. True Positives (TPs), False Positives (FPs) and Positive Predictive Value (PPV) arising from our different models (Human1, Human1-O1, Human1-O2, Human1-O3, Human-D1, Human-D2, Human-D3, Human1-T1, Human1-T2, Human1-T3) using the essentiality data presented in DepMap. In the boxplots, the center line represents the median, the bounds of the box correspond to the 25th and 75th percentiles, the whiskers indicate the interquartile range by 1.5 times and the circles are the outliers.

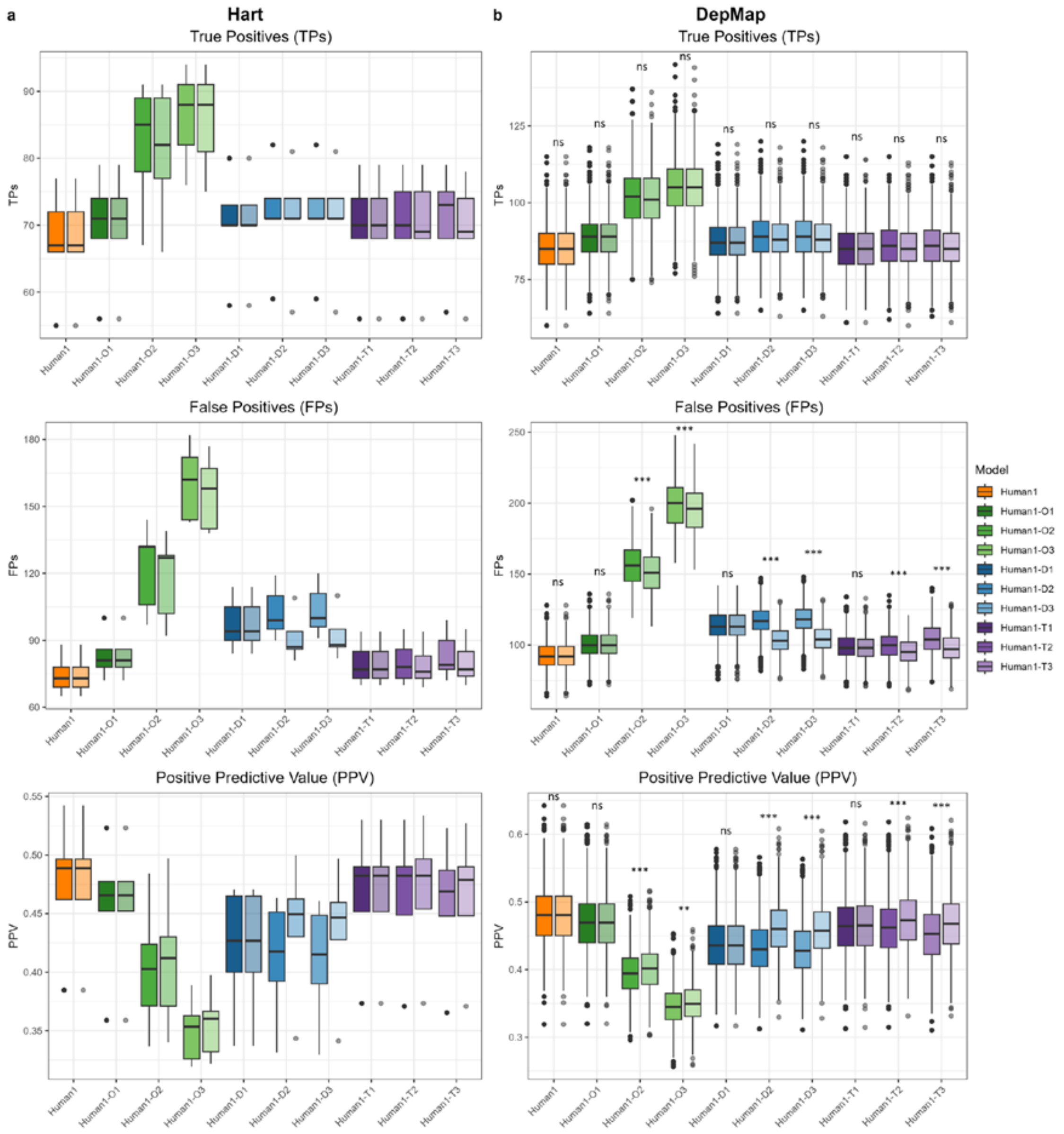

Supplementary Figure 8. Impact of adaptation pathways in gene essentiality analysis in Human1 and different integrated models. For each model, the boxplot on the left corresponds to the essentiality results without considering adaptation, while the boxplot on the right corresponds to the essentiality results after considering adaptation. In the boxplots, the center line represents the median, the bounds of the box correspond to the 25th and 75th percentiles, the whiskers indicate the interquartile range by 1.5 times and the circles are the outliers. Note: ***/ns refer to the statistical significance level from an unpaired one-sided Wilcoxon test, respectively ***: p-value<=0.001 and ns: non-significant.

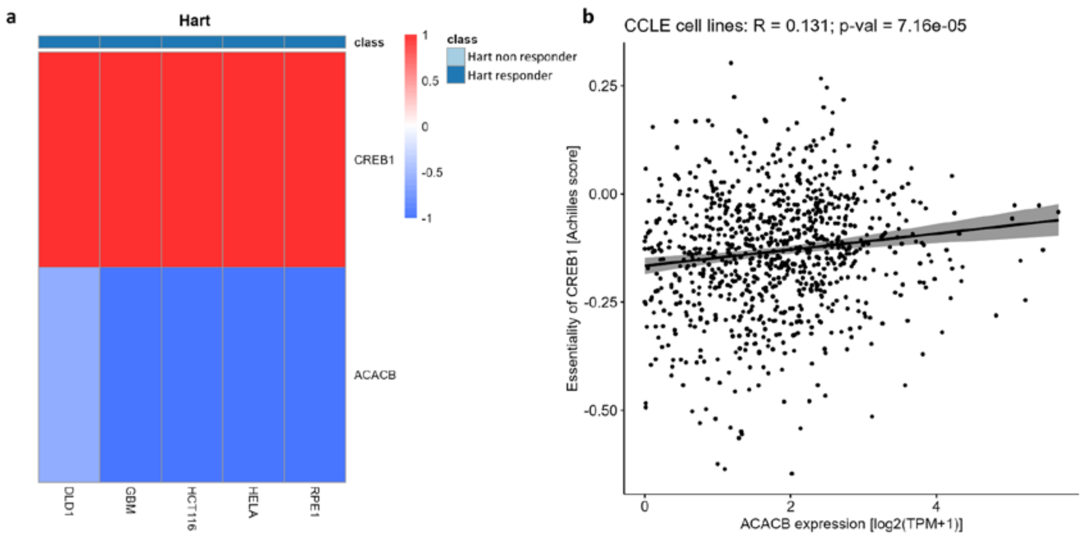

Supplementary Figure 9. Prediction of essentiality of CREB1 in Hart2015 and correlation analysis in DepMap cell lines. a) Expression of the genes CREB1 and ACACB, which comprise a new gMCS that is predicted in Human1-T1. CREB1 is predicted essential in all the cell lines of Hart2015 because ACACB is not expressed. b) Correlation between the essentiality of CREB1 (CRISPR knockout screen data from DepMap) and the expression of ACACB in log2(TPM+1). Pearson correlation coefficient *r* and its associated one-sided correlation test *p*-value are indicated. Note here that multiple hypothesis correction is not applicable here. Linear regression line and their associated 95% confidence interval is shown (shaded area).

a

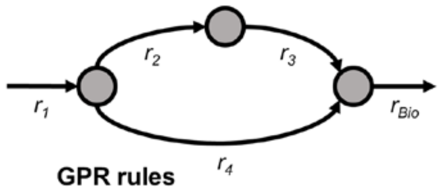

GPR rules

$r_1: g_1$
$r_2: g_2$
$r_3: g_2$
$r_4: (g_2 \,\&\, (g_3|g_4))|g_5$

Regulatory information

Source	Target	Interaction
g_5	g_2	+
g_7	g_2	+
g_7	g_3	-
g_6	g_4	+
g_7	g_4	+
g_8	g_5	+
g_9	g_7	-
g_4	g_8	+
g_{10}	g_8	+
g_6	g_{10}	+
g_9	g_{10}	+

b

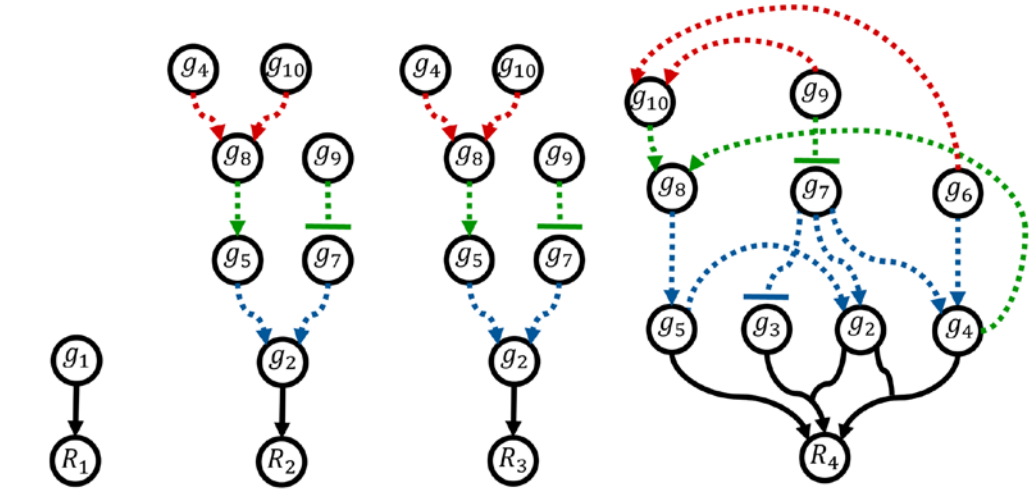

eGPR rules

$R_1 = g_1$

eGPR rules

$R_2 = g_2$
$g_2 = g_5|g_7$
$g_5 = g_8$
$g_7 = !g_9$
$g_8 = g_4 \mid g_{10}$

eGPR rules

$R_3 = g_2$
$g_2 = g_5|g_7$
$g_5 = g_8$
$g_7 = !g_9$
$g_8 = g_4 \mid g_{10}$

eGPR rules

$R_4 = (g_2 \,\&\, (g_3|g_4))|g_5$
$g_2 = g_5| g_7$
$g_3 = !g_7$
$g_4 = g_6 \mid g_7$
$g_5 = g_8$
$g_7 = !g_9$
$g_8 = g_4| g_{10}$
$g_{10} = g_6| g_{10}$

Supplementary Figure 10. Example network in Figure 3 with 1, 2 and 3 regulatory layers. a) Example network in Figure 3 and associated regulatory information. b) Systematic addition of regulatory information. Arrows in blue represent the first regulatory layer; arrows in green represent the second regulatory layer and arrows in red represent the third regulatory layer.

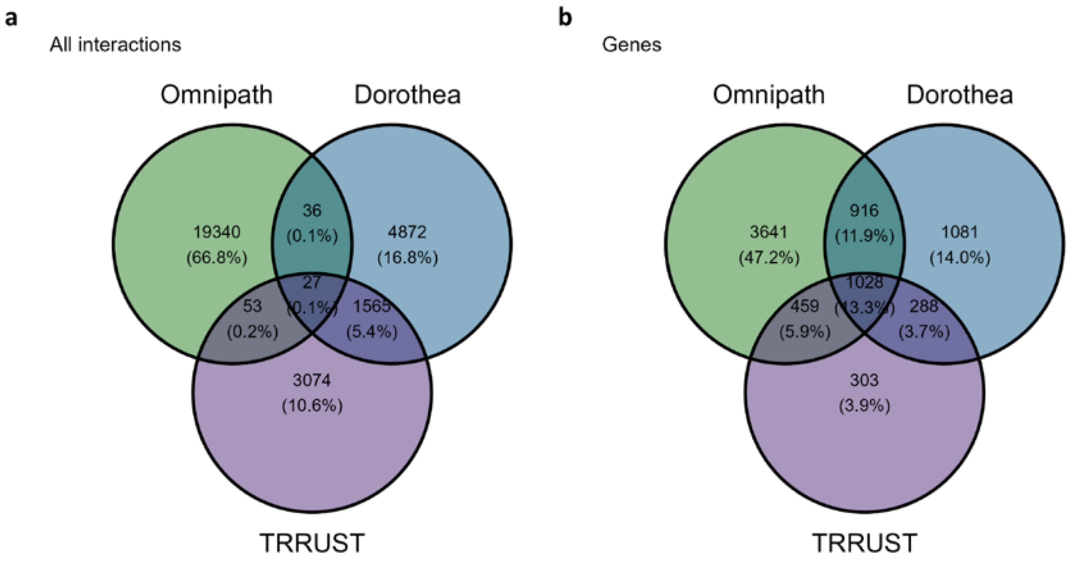

Supplementary Figure 11. Overlap between different regulatory network databases. a) Venn diagram of the genetic interactions annotated in the 3 regulatory databases analyzed: Omnipath, Dorothea and TRRUST; b) Venn diagram of the genes present in the 3 regulatory databases.

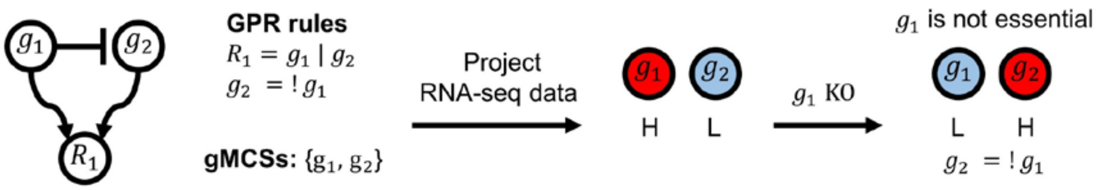

Supplementary Figure 12. Example integrated network and adaptation mechanism upon gene knockout. In this simple network, we only have on gMCS: {g1, g2}. After RNA-seq data projection, g1 is a potential essential gene because g2 is lowly expressed in that sample. However, the knockout of g1 implies the activation of g2, and thus, the essentiality of g1 is discarded.

Supplementary Tables

Supplementary Table 1. Summary of single-layer integrated models combining different regulatory databases and computed gMCSs. Results correspond to models integrating 2 or the 3 regulatory databases. Computation time is given in seconds (s). Abbreviations: 'Human1-D∩T1': integrated model with Human1 and the intersection of Dorothea and TRRUST; 'Human1-O∩T1': integrated model with Human1 and the intersection of Omnipath and TRRUST; 'Human1-O∩D1': integrated model with Human1 and the intersection of Omnipath and Dorothea; 'Human1-O∩D∩T1': integrated model with Human1 and the intersection of Omnipath, Dorothea and TRRUST; 'Human1-D∪T1': integrated model with Human1 and the union of Dorothea and TRRUST; 'Human1-O∪T1': integrated model with Human1 and the union of Omnipath and TRRUST; 'Human1-O∪D1': integrated model with Human1 and the union of Omnipath and Dorothea; 'Human1-O∪D∪T1': integrated model with Human1 and the union of Omnipath, Dorothea and TRRUST.

Model	Number of genes	*G* matrix dimension	*G* matrix computation time (s)	Simplified G matrix	Number of gMCSs (length ≤ 5)
**Human1-D∩T1**	2,486	2,109x11,573	1,066	1,741x11,573	10116 (25)
**Human1-O∩T1**	2,433	1,787x11,573	569	1,613x11,573	10091 (0)
**Human1-O∩D1**	2,430	1,799x11,573	582	1,615x11,573	10111 (20)
**Human1-O∩D∩T1**	2,426	1,774x11,573	647	1,605x11,573	10091 (0)
**Human1-D∪T1**	2,675	6,320x11,573	6,091	2,013x11,573	11150 (1059)
**Human1-O∪T1**	3,235	3,556x11,573	3,955	1,898x11,573	11766 (1675)
**Human1-O∪D1**	3,117	5,322x11,573	5,554	2,078x11,573	14716 (4625)
**Human1-O∪D∪T1**	3,254	8,251x11,573	15,665	2,056x11,573	12060 (1969)

Supplementary Table 2. Summary list of the new synthetic lethals and context-specific essential genes obtained with Human1-T1 (single-layer integrated model with Human1 and TRRUST) and Human1-T2 (double-layer integrated model with Human1 and TRRUST).

Gene	Gene type	gMCS	Model	Cell line	Literature
E2F1	Regulatory	{E2F1}	Human-T1<br>Human-T2	ALL	Wu *et al.*[1]
KLF5	Regulatory	{KLF5}	Human-T1<br>Human-T2	ALL	Dong and Chen[2]<br>Takagi *et al.*[3]
NR1H4	Regulatory	{NR1H4}	Human-T1<br>Human-T2	ALL	Lee *et al.*[4]
SP1	Regulatory	{SP1}	Human-T1<br>Human-T2	ALL	Vizcaíno *et al.*[5]<br>Zhao *et al.*[6]<br>Lee *et al.*[7]
SREBF2	Regulatory	{SREBF2}	Human-T1<br>Human-T2	ALL	Wen *et al.*[8]
CREB1	Regulatory	{CREB1, ACACB}	Human-T1<br>Human-T2	DLD1,<br>GBM,<br>HCT116,<br>HELA,<br>RPE1	Fang *et al.*[9]<br>Yang *et al.*[10]
CTPS2	Metabolic	{CTPS2, TWIST1, TWIST2}	Human-T1	HELA	
PISD	Metabolic	{PISD, SPHK2, SPHK1, HLF}	Human-T1<br>Human-T2	HCT116	
		{PISD, SPHK2, BTG2, HLF}	Human-T1<br>Human-T2	HELA	Bellance *et al.*[11]
RARA	Regulatory	{RARA, SCD5}	Human-T1<br>Human-T2	HELA	
RPIA	Metabolic	{RPIA, HIPK2, XYLB, NFATC1, ADA}<br>{RPIA, XYLB, NFATC1, FOXO4, ADA}	Human-T2	DLD1	Qiu *et al.*[12]
		{RPIA, XYLB, NFATC1, FOXO4, ADA}		RPE1	
STAT6	Regulatory	{STAT6, CYP2R1, NFATC1, CYP27A1}	Human-T1	RPE1	
TXN2	Metabolic	{TXN2, PPARD}	Human-T1	HELA	Zhang *et al.*[13]

Supplementary References

1. Wu, L. *et al.* The E2F1–3 transcription factors are essential for cellular proliferation. *Nature* **414**, 457–462 (2001).

2. Dong, J. T. & Chen, C. Essential role of KLF5 transcription factor in cell proliferation and differentiation and its implications for human diseases. *Cellular and Molecular Life Sciences* **66**, 2691–2706 (2009).

3. Takagi, Y. *et al.* High expression of Krüppel-like factor 5 is associated with poor prognosis in patients with colorectal cancer. *Cancer Sci* **111**, 2078–2092 (2020).

4. Lee, Y. J. *et al.* The Role of Nuclear Receptor Subfamily 1 Group H Member 4 (NR1H4) in Colon Cancer Cell Survival through the Regulation of c-Myc Stability. *Mol Cells* **43**, 459 (2020).

5. Vizcaíno, C., Mansilla, S. & Portugal, J. Sp1 transcription factor: A long-standing target in cancer chemotherapy. *Pharmacol Ther* **152**, 111–124 (2015).

6. Zhao, Y. *et al.* Inhibition of the transcription factor Sp1 suppresses colon cancer stem cell growth and induces apoptosis in vitro and in nude mouse xenografts. *Oncol Rep* **30**, 1782–1792 (2013).

7. Lee, J. H., Park, T. H. & Rhee, W. J. Inhibition of Apoptosis in HeLa Cell by Silkworm Storage Protein 1, SP1. *Biotechnology and Bioprocess Engineering* **20**, 807–813 (2015).

8. Wen, Y. A. *et al.* Downregulation of SREBP inhibits tumor growth and initiation by altering cellular metabolism in colon cancer. *Cell Death Dis* **9**, 265 (2018).

9. Fang, Z. *et al.* CREB1 directly activates the transcription of ribonucleotide reductase small subunit M2 and promotes the aggressiveness of human colorectal cancer. *Oncotarget* **7**, 78055 (2016).

10. Yang, Q., Yu, W. & Han, X. Overexpression of microRNA-101 causes anti-tumor effects by targeting CREB1 in colon cancer. *Mol Med Rep* **19**, 3159–3167 (2019).

11. Bellance, N. *et al.* Doxorubicin Inhibits Phosphatidylserine Decarboxylase and Modifies Mitochondrial Membrane Composition in HeLa Cells. *Int J Mol Sci* **21**, 1317 (2020).

12. Qiu, Z. *et al.* MicroRNA-124 Reduces the Pentose Phosphate Pathway and Proliferation by Targeting PRPS1 and RPIA mRNAs in Human Colorectal Cancer Cells. *Gastroenterology* **149**, 1587-1598.e11 (2015).

13. Zhang, X. *et al.* Disruption of the mitochondrial thioredoxin system as a cell death mechanism of cationic triphenylmethanes. *Free Radic Biol Med* **50**, 811–820 (2011).
